# Fundamentals to Apply Magnetic Nanoparticles for Hyperthermia Therapy

**DOI:** 10.3390/nano11051203

**Published:** 2021-05-01

**Authors:** Hira Fatima, Tawatchai Charinpanitkul, Kyo-Seon Kim

**Affiliations:** 1 Department of Chemical Engineering, Kangwon National University Chuncheon, Kangwon-do 24341, Korea; hirafatima13@kangwon.ac.kr; 2 Center of Excellence in Particle Technology, Department of Chemical Engineering, Faculty of Engineering, Chulalongkorn University, Bangkok 10330, Thailand; ctawat@chula.ac.th

**Keywords:** cancer, magnetic hyperthermia, magnetic nanoparticles, saturation magnetization, specific absorption rate

## Abstract

The activation of magnetic nanoparticles in hyperthermia treatment by an external alternating magnetic field is a promising technique for targeted cancer therapy. The external alternating magnetic field generates heat in the tumor area, which is utilized to kill cancerous cells. Depending on the tumor type and site to be targeted, various types of magnetic nanoparticles, with variable coating materials of different shape and surface charge, have been developed. The tunable physical and chemical properties of magnetic nanoparticles enhance their heating efficiency. Moreover, heating efficiency is directly related with the product values of the applied magnetic field and frequency. Protein corona formation is another important parameter affecting the heating efficiency of MNPs in magnetic hyperthermia. This review provides the basics of magnetic hyperthermia, mechanisms of heat losses, thermal doses for hyperthermia therapy, and strategies to improve heating efficiency. The purpose of this review is to build a bridge between the synthesis/coating of magnetic nanoparticles and their practical application in magnetic hyperthermia.

## 1. Introduction

During the early 21st century, the treatment of cancer has been considered the most challenging health issue. Despite intensive advances in clinical technology, cancer is still the leading cause of death all over the world [1,2,3,4]. Cancer is known to develop cell signaling and apoptosis, making it a highly complex and incompressible disease [5,6,7]. The principal types of cancer therapies include radiation therapy, chemotherapy, and surgery [8,9]. An additional treatment modality, hyperthermia, involves heating the tumor region with significant damage to normal cells [10]. Hyperthermia treatment is divided into three main types: whole-body hyperthermia, regional hyperthermia, and local hyperthermia [3]. In whole-body hyperthermia, the whole body is exposed to heat by an external heat source, such as radiofrequency waves, microwaves, or ultrasound waves. This treatment method can lead to bad side effects due to non-selective heating [11]. Regional hyperthermia is an advanced method of hyperthermia treatment, which heats a selected large area of cells, such as an organ or limb or body cavity. Regional hyperthermia requires external applicators or thermal perfusions during therapy [12]. Local hyperthermia is more often used to kill cancer cells in a selected small area, with greater selectivity by introducing heat carriers into the body, such as Fe, Co, and Ni metallic nanoparticles and their oxides, which act as a heat source [13]. Given the above-mentioned challenges with hyperthermia treatment, a novel method with improved effectiveness must be developed to treat cancer. In this regard, scientific research is directed towards the application of magnetic nanoparticles (MNPs) as a source of heat, called magnetic hyperthermia [14].

Magnetic hyperthermia involves heating at a sustained temperature above 43 °C, which causes the necrosis of cancerous cells, which are more sensitive to heat than healthy cells. Thus, magnetic hyperthermia is a promising technique for treating cancer compared to other heating techniques [10]. Magnetic hyperthermia involves the administration (intercellular or transcellular) of MNPs followed by the application of an alternating external magnetic field, which generates heat within the tumor area [15,16,17]. However, it should be considered that less than 1% of an intravenous administered dose reaches the tumor [18].

**Figure 1:**
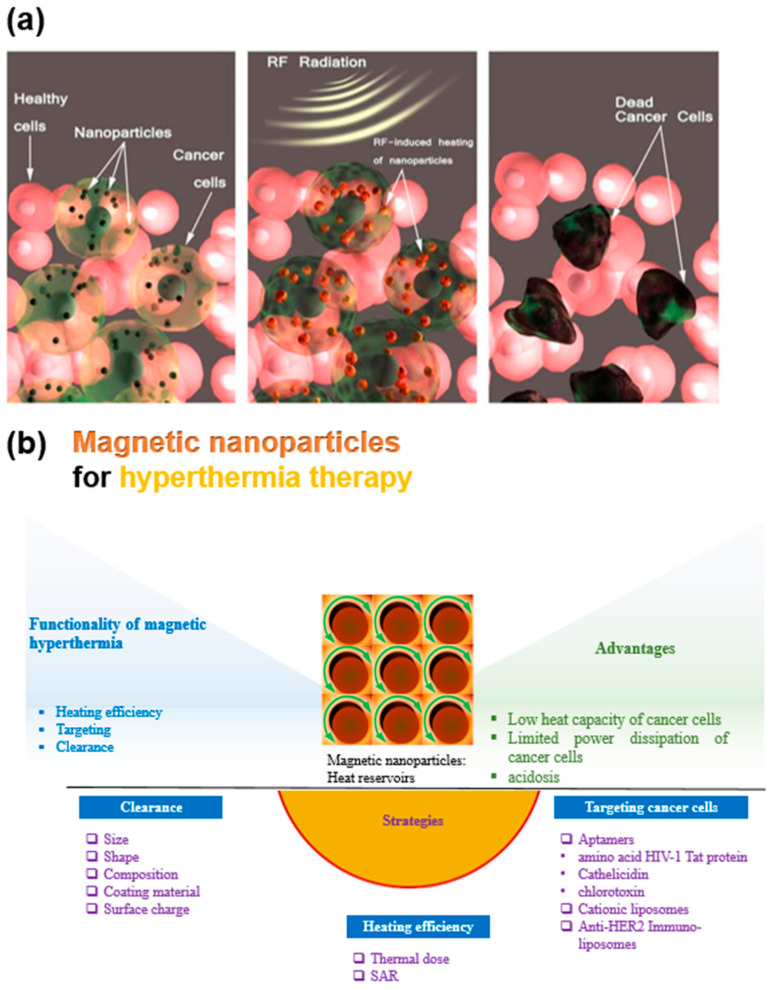
(**a**) Schematic representation of the magnetic hyperthermia treatment procedure. Reprinted with permission from [21]. Copyright 2014 Springer Nature. (**b**) Factors affecting the functionality of magnetic hyperthermia.

Generally, blood flow decreases with the growth of cancer cells because of the progressive deterioration of vascular beds and the rapid growth of cancer cells. Consequently, the heat dissipation by blood flow in cancer cells is slower compared with normal tissues. Moreover, the heat capacity of cancer cells appears to be lower than that of normal tissues. Thus, with the limited power dissipation and lower heat capacity of the tumor area, the temperature of cancer cells becomes higher than that of normal tissues [19], which expedites the apoptosis of cancer cells [20], as shown in Figure 1a [21]. Cancer cells also face the deprivation of nutrients and oxygen due to disorganized vasculature [22]. Moreover, hyperthermia also leads to acidosis in the regions of hypoxia by heat exposure [23]. All these factors make the cancer cells more sensitive to heat. Hyperthermia treatment may decrease or increase the oxygenation of cancer cells depending on the exposure temperature and time [24]. MNPs can effectively cross the blood–brain barrier, which has been found to be an essential step in treating brain cancer [25]. MNPs can also be coupled with biological molecules, such as proteins (5–50 nm), viruses (20–450 nm), and genes (10–100 nm long) [26] to facilitate targeted therapy. 

The proper functionality of MNPs in magnetic hyperthermia depends on several factors, such as heating efficiency, targeting, and clearance of MNPs (Figure 1b). The heating efficiency can be described in terms of thermal dose [27] and specific absorption rate (SAR) [28]. For minimization of the thermal dose required for sufficient heat, development of MNPs with high SAR is needed [8]. Furthermore, functional moieties are named as receptors (magnetic cationic liposomes (MCLs) [29,30], immunoliposomes [30], aptamers [31,32], or peptides [33,34,35]), which help to facilitate active targeting of cancer, and thus enhancing the cancer cell killing rate. Clearance of MNPs through reticuloendothelial circulation is another prominent factor affecting the heating efficiency that is dependent on the size, shape, and surface charge of the MNPs. Basically, the standardization of MNPs for magnetic hyperthermia is a necessary prerequisite. As a result, many key laboratories in the national metrology institutes of many countries have tried to demonstrate proficiency in SLP measurement via interlaboratory comparisons using reference materials. Accordingly, consolidated procedures for SLP measurement on calibrated instrumentations under designated quality assurance have been documented internationally [36].

Over the past decade, several review papers have been published discussing the synthesis, magnetic properties, functionality, and biomedical applications of MNPs. The main objective of this manuscript is to introduce the basics of magnetic hyperthermia and types of heat losses during the heating process to highlight the role of heating efficiencies such as thermal dose and SAR. Moreover, targeting receptor moieties facilitates the efficient selective killing of cancer cells. Several methods for the synthesis of small-sized MNPs and biocompatible coatings have been introduced to link with typical examples of magnetic hyperthermia applications and the circulation rate-retention time of MNPs. Typical case studies for the in vivo application of MNPs in magnetic hyperthermia are also summarized.

## 2. Mechanism of Magnetic Hyperthermia Therapy

In principle, MNPs are introduced into the human body by injecting a solution containing a calculated amount of MNPs. To produce heat in the human body, MNPs are subjected to an external alternating magnetic field generated by radio frequency (RF) induction coils. Induction coils surround the biological object externally and produce agitation of MNPs [37]. The resulting electromagnetic energy is transferred to the neighborhood cells in the form of heat, which raises the temperature of cancer cells more than non-cancer cells. The temperature difference between cancer cells and non-cancer cells can reach 2 to 3 °C. The magnetic field generated by the induction heating coils penetrates deep into the tissues, such as subcutaneous fat, without excessive damage to healthy tissues [14]. These external applicators are typically operated at frequencies of 13.56, 27.12, and 40 MHz, with a depth of a few centimeters for penetration. To improve heating efficiency, magnetic materials may be injected into the targeted area [38]. Tamarove et al. [21] reported the effect of RF-induced heating of nanoparticles in aqueous colloidal solutions. The MNPs are first selectively collected in tumor areas through selective targeting. Then, a magnetic field is generated to produce heat, causing a local increase of temperature in the tumor area and selective death of cancerous cells, while adjacent healthy tissues remain unaffected.

When MNPs are exposed to alternating magnetic fields four different mechanisms can function to generate heat within the therapeutic system, named as (1) hysteresis [39], (2) eddy current [40,41], (3) Néel or Brownian relaxation [40], and (4) frictional losses [42]. However, Néel or Brownian relaxation is dominant in superparamagnetic MNPs [43]. The magnetic moment’s oscillation causes the displacement of domain walls, which generates heat. Upon removal of the magnetic field, the magnetic moments relax, either by the individual magnetic moment within the particles, named Néel relaxation, or by the rotation of individual particles around their axis, named Brownian relaxation.

## 3. Heating Efficiency of MNPs

The successful application of magnetic hyperthermia requires the administration of stable biocompatible nanoparticles of moderate size/shape at a modest dose. Sapareto et al. [27] proposed cumulative equivalent minutes (CEMs) at 43 °C as a model to calculate a thermal dose. A reference temperature of 43 °C has been selected arbitrarily to convert all thermal exposure to “equivalent-minutes” at this temperature, using the Equation (1):

(1) $$t_{43}=\overset{t=\mathrm{final}}{\underset{t=0}{\sum }}R^{\left(43-\;\bar{T}\right)}\Delta t$$

where $t_{43}$ is the equivalent time at temperature 43 °C, $\;\bar{T}$ is the average temperature during time Δt. R = 0.5 above 43 °C and R = 0.25 below 42 °C [27]. 

The development of MNPs presenting high SAR values is needed to minimize the necessary thermal dose. SAR is the parameter for measuring the power deposition and heating efficiency of MNPs [8]. The experimental setup to calculate SAR is simple in configuration. Typically, it involves the suspension of a known concentration of material in a liquid of known heat capacity over a period of time. The experimental setup is then exposed to an alternating magnetic field of known strength and frequency, and the temperature change is recorded continuously over a period of time [44]. Generally, SAR is defined as the efficiency of MNPs to transform magnetic energy into heat, which is expressed by the Equation (2) [45]:

(2) $$\mathrm{SAR}\;=\frac{\mathrm{absorbed}\;\mathrm{power}}{\mathrm{mass}\;\mathrm{of}\;\mathrm{MNPs}}$$

There are two main strategies for SAR measurement, which are classified as calorimetric and magnetometric methods. According to the calorimetric method, if the time progression of sample temperature is documented, the time derivative of temperature at t = 0 can be attained. Subsequently, the SAR value of the sample can be calculated by using Equation (3):

(3) $$\mathrm{SAR}=\frac{C_{p,s}}{m_{\mathrm{MNPs}}}{\left|\frac{\mathrm{dT}}{\mathrm{dt}}\right|}_{t=0}$$

where $C_{p,s}$ is the heat capacity of the sample and $m_{\mathrm{MNPs}}$ is the mass of the MNPs. Many factors have been reported in the literature affecting the accuracy of calorific SAR values, such as sample volume, magnetic field gradient, thermal loss, and measurement methodology [46,47]. Fast heating leads to a high-temperature gradient when dealing with a large volume of the sample, due to high heat dissipation, thus SAR accuracy has shown an ultimate dependency on temperature sensor positioning [47]. In another study, a sample with a high magnetic values reduced the intensity of the applied field, and the measured SAR values were found to be underestimated [46]. Moreover, the SAR value measurement is also dependent on the initial value slope, and the linear slope region is always limited, except for in adiabatic processes [48]. 

On the other hand, magnetometric methods for SAR measurement consist of measuring the dynamic magnetization ${\;\vec{M}}_{t}$ of the sample and then taking the integration of ${\;\vec{M}}_{t}$ versus applied magnetic field ${\;\vec{M}}_{t}$, by using the following Equation (4) [49]:

(4) $$\mathrm{SAR}=\frac{f}{c}\oint^{\;}{\;\vec{M}}_{t}.{d\vec{H}}_{t}$$

where f is the frequency and c is the weight concentration of MNPs. 

For clinical applications of MNPs, two parameters are required to be considered: applied frequency of magnetic field and the optimum applied AC magnetic field. The heating effect of MNPs is an increasing function of both frequency and field amplitude [39]. However, a limit comes with the cost of increasing frequency and high field. For instance, Hergt et al. [39] summarized that a frequency of 500 kHz and a field amplitude of 10 kA m^−1^ is appropriate for magnetic hyperthermia. In another study, Hergt et al. [50] suggested that a 400 kHz magnetic field frequency for field amplitudes up to 11.7 kA m^−1^ is appropriate for magnetic hyperthermia application. Dutz et al. [51] proposed a field amplitude of about 10 kA m^−1^ and frequency of about 400 kHz for the treatment of breast cancer. Considering the particular characteristics of MNPs, such as size, crystal structure, shape, magnetic susceptibility, and saturation magnetization, along with the frequency (f) and applied strength (H), Liu et al. [52] suggested the following formula for the calculation of SAR values;

(5) $$\mathrm{SAR}=\frac{8\pi^{3}{\mu_{0}}^{2}{M_{s}}^{2}r^{3}{H_{0}}^{2}f^{2}\tau }{3{\rho k}_{B}T{\left(1+2\pi f\tau \right)}^{2}}$$

where $\mu_{0}$ = 4π × 10^−7^ N A^−2^ is the permeability of free space, ρ is the density of the MNP, and H_0_ is the applied magnetic field. We can see that the SAR in Equation (5) depends on the value of H_0_ × f, as suggested by Hergt et al. [39].

One important parameter during in vivo magnetic hyperthermia application is the aggregation/dispersion of MNPs. The MNPs show a much enhanced heating performance when the dispersion is more uniform compared with aggregated MNPs. This decreased heating performance is related to both, immobilization of MNPs blocking the Brown relaxation mechanisms, and increase in the dipolar interaction of MNPs [53]. Thus measurement of heating efficiency with immobilized particles with a blocked Brown relaxation mechanism is suggested, as it does not contribute to SAR values in these conditions [54]. Iacovita et al. [55,56] conducted experiments to evaluate the heating potential in highly viscous PEG1k or in solid PEG8k, indicating that immobilization of MNPs led to a substantial decrease in SAR values.

Upon immersion of MNPs within the biological media, their surfaces start to be covered with biological molecules by the interaction of serum proteins and MNPs, leading to the formation of a nanoparticle–protein complex, named protein corona. Protein corona has been found to be another important factor affecting the in vivo heating efficiency of MNPs by altering the inherent properties of MNPs [57]. The development of protein corona is dependent on many factors, including the physicochemical properties of MNPs, the local temperature at the surface of MNPs, and the temperature and time of incubation [58]. The interaction between the protein and MNPs results in the alteration of protein functionality, biocompatibility, and aggregation, and thus producing potential immunogenicity. Therefore, an understanding of protein–MNP interactions and protein corona retention time determination is needed to design functional and safe MNPs for magnetic hyperthermia [59].

**Figure 2:**
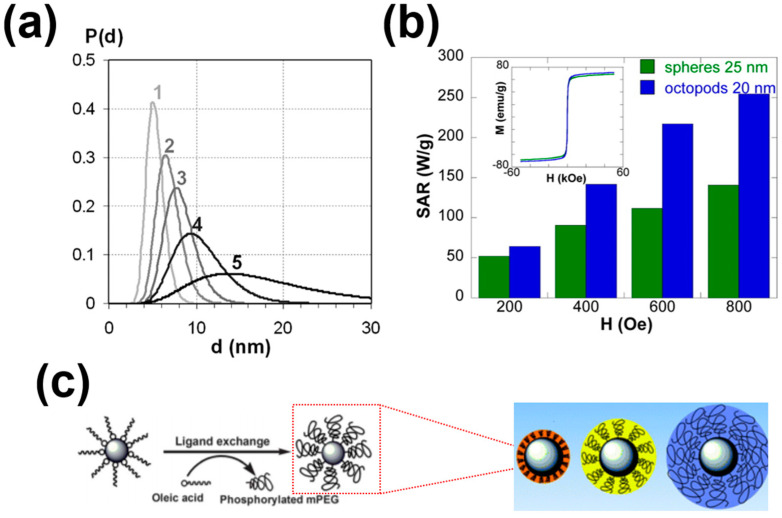
(**a**) Size distributions versus magnetization curves of five different samples Reprinted with permission from [68]. Copyright 2007 American Chemical Society. (**b**) SAR values for nano-octopods and spherical nanoparticles, and inset is the M–H loops at room temperature Reprinted with permission from [73]. Copyright 2016 American Chemical Society. (**c**) A schematic diagram of magnetic cores with different molecular weights of PEG coating. Reprinted with permission from [90]. Copyright 2012 The Royal Society of Chemistry.

Significant research has been carried out focusing on improvements in SAR by preparing suitable magnetic materials by controlling their size, shape, composition, and coating [60,61]. Table 1 summarizes the SAR values of coated/uncoated MNPs. For an efficient therapeutic technique, MNPs must be stable at pH 7 in the physiological environment, which is dependent on the particle size and surface charge chemistry [62]. MNPs have an efficient potential in localized heating of cells, due to their size-dependent properties [63]. In addition, the size of MNPs is a crucial factor for determining their uptake by targeted cells and elimination from the body. For example, MNPs of a size larger than 200 nm are easily absorbed by the spleen and liver, while particles smaller than 10 nm are rapidly removed via renal clearance [64]. The Ms of MNPs decreases rapidly with a reduction in size, due to reduction in magnetic anisotropy, while larger sized MNPs show high Ms [65]. Thus large-sized MNPs are favorable, as the Ms values are proportional to SAR [66,67] and inversely proportional to the size distribution (Figure 2a) [68].

Shape is another parameter that has a significant influence on heating performance. Song et al. [69] reported that under the same Fe concentration and 100 kHz/30 kA/m magnetic field, quasi-cubical showed a superior SAR compared with spherical Fe_3_O_4_ nanoparticles. In another study, an identical size/composition of Zn_0.4_Fe_2.6_O_4_ nanocubes showed significantly high SAR than spherical nanocubes, due to the higher disordered spin of cubic material leading to high Ms (165 emu/g) [70]. Similar results were also presented by Martinez-Boubeta et al. [71], whereby it was shown that cubic iron oxide nanoparticles exhibited higher SAR than spherical nanoparticles. Serantes et al. [72] reported an enhanced heating performance of a chain-like magnetotactic bacteria compared with a random orientation system. Recently, Nemati et al. [73] reported the dominant heating performance of nano-octopods compared with spherical particles, as shown in Figure 2b.

**Table 1:** SAR values for MNPs from the literature.

No.	Material	Coating	Field KA/m	Frequency kHz	SAR W/g	Ref.
1	Fe_2_O_3_	N/A	12.5	500	626	[5]
2	Iron oxide	N/A	N/A	110	322	[67]
3	Iron oxide	N/A	500	15.5	716	[74]
4	Iron oxide	N/A	30	210	702	[75]
5	FePt@Fe_3_O_4_	N/A	630	18.8	1120	[76]
6	CoFe_2_O_4_	N/A	24.8	700	360	[68]
7	Fe_0.6_Mn_0.4_O	N/A	366	32	535	[77]
8	Zn_0.4_Mn0_.6_Fe_2_O_4_	N/A	500	3.7	432	[78]
9	Magnetosome	N/A	10	410	960	[79]
10	Magnetosome	N/A	23.9	765	1200	[72]
11	Iron oxide	Dextran	12.5	500	625	[80]
12	Iron oxide	CTAB	63	358	2483	[81]
13	Iron oxide	CTAB	47.8	488	5000	[82]
14	Iron oxide	PEG	29	520	2452	[83]
15	Iron oxide	PEG	29	520	2452	[84]
16	Iron oxide	GO	32.5	400	5160	[85]
17	Iron oxide	mPEG	35	400	2213	[86]
18	CoFe_2_O_4_	PMAO	32	105	915	[87]
19	CoFe_2_O_4_ @MnFe_2_O_4_	DMSA	37.3	500	2250	[88]
20	CoFe_2_O_4_ @Zn_0.4_Fe_2.6_O_4_	DMSA	37.4	500	10600	[70]
21	MnFe_2_O_4_	GO	60	240	1588	[89]

Note: Cetyl trimethylammonium bromide: CTAB; Polyethylene glycol: PEG; Graphene oxide: GO; Poly maleic anhydride 1−octadecene: PMAO; dimercaptosuccinic acid: DMSA; N/A: Not available.

The coating can have a dominant effect on the heat efficiencies of MNPs. Generally, it is assumed that the surface properties of MNPs are more important compared with the core properties, due to the direct connectivity of surface materials with the biological environment. Moreover, hydrophobic surfaces with a large surface-to-volume ratio force them to be agglomerated to form clusters [3]. Hence, it is necessary to protect the MNPs surface with biocompatible coating materials. Complete coating of MNPs with a complete conductor, e.g., SiO_2_, reduces the outflow of heat, hence decreasing the heating efficiency. Furthermore, the coating thickness also alters the heating rates [90]. Various coating materials have been developed, such as polymeric, organic, and inorganic stabilizers and targeting ligands [91,92]. For biomedical applications, several polymeric coating materials (PCM) are potentially applicable [93]. The commonly used PCMs are listed in Table 2. PCMs enhance colloidal stability and, in turn, increase their time circulating in the blood. In addition, PCMs prevent coagulation by improving biocompatibility. However, PCMs result in large-sized MNPs, and this is why researchers are trying to stabilize nanoparticles using non-polymeric materials. Moreover, the molecular weight of the surface coating also contributes to the modulation of SAR values. Liu et al. [90] developed three different surface coating molecular weights, named poly(ethylene glycol) methyl ether (Figure 2c). Their results demonstrated a highest SAR of 930 W/g, which is a 2.5-fold increase, with the decrease in the molecular weight of the surface coating from 5000 to 2000. Oleic acid, lauric acid, and stearic acid dodecyl phosphonic acid are typical examples of non-polymeric organic coating materials [94]. Inorganic materials such as silica help in binding with various biological molecules and with the stability of MNPs in the biological solution [95]. 

## 4. Targeting Cancer Cells with MNPs

**Figure 3:**
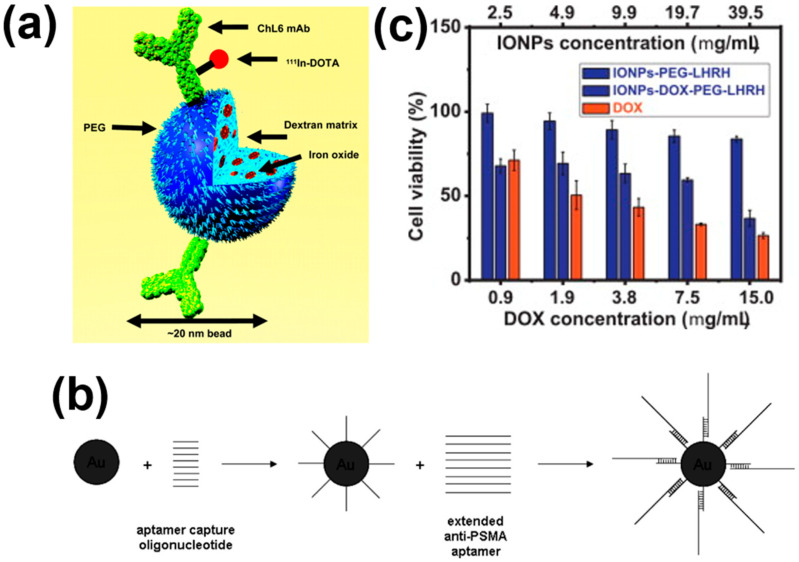
(**a**) Schematic diagram for the conjugation of ChL6 antibody with MNPs. Reprinted with permission from [108]. Copyright 2007 SNMMI. (**b**) Schematic representation for the conjugation of anti-PSMA aptamers with gold nanoparticles. Reprinted with permission from [31]. Copyright 2008 American Chemical Society. (**c**) The cell viability of A2780/AD human ovarian cancer cells using a DOX, IONPs-DOX-PEG-LHRH, and DOX loaded IONPs-DOX-PEG-LHRH system. Reprinted with permission from [34]. Copyright 2013 Elsevier B.V.

Intravenously injected MNPs accumulate within the tumor area due to their endoplasmic reticulum (EPR) effect [104]. The accumulation rate of MNPs can be further enhanced with the targeting of cancer cells using antibodies or other ligands [44]. Cancer cells have special receptors that can be targeted through functional moieties present over the surfaces of MNPs. These receptors are produced due to their mutated genotype, and hence can be distinguished from healthy cells. Identification of these cancer-specific receptors helps in the targeting of only cancer cells for hyperthermia treatments and allows treatment of tumors with minimal therapy-related toxicity [105]. MNP conjugated antibodies have been intensively investigated and delivered to tumor-specific antigens, such as PSMA [106], HER-2 [107], and L6 [108] for magnetic hyperthermia. DeNardo et al. [108] conjugated the ChL6 antibody with MNPs to target tumor associated L6 antibody (Figure 3a) to achieve an enhanced accumulation rate of MNPs. Despite the potential increased accumulation of MNPs with antibody–antigen interaction, the conjugation process faces a limitation in terms of stability and conjugation chemistry. For example, MLN2704 antibody significantly enhanced toxicity and limited magnetic hyperthermia activity due to deconjugation processes [109]. 

To overcome this limitation of the antibody–antigen system, MNPs can also be conjugated with magnetic cationic liposomes (MCLs) [29,30], aptamers [31,32], or peptides [33,34,35]. Kawai et al. [29] employed magnetic cationic liposomes (MCLs) to treat glioma cancer after heating to 45 °C. Induction of anticancer immunity was also demonstrated for solid T-9 rat glioma tissues using MCLs. Treated cancer cells disappeared completely in glioma containing rats exposed to an alternating magnetic field of 118 kHz, 384 Oe. The treatment was repeated three times for 30 min each, at 24-h intervals. They also reported that the cancer cells where MCLs were not injected also disappeared completely, due to the generation of antitumor immunity. Ito et al. [30] developed anti-HER2 Immuno-liposomes@MNPs to treat SKBr3 breast cancer cells. They demonstrated a 60% incorporation of MNPs into the cancer cells resulting in potential cytotoxicity. In another study, dextran-coated MNPs were incorporated into the liposome to target AH60C rat tumors after heating to 42 °C. Treated cells disappeared completely by exposing them to 500 kHz for 7 min. A significantly enhanced survival rate of tumor cells was also recorded compared with controls [110]. 

Javier et al. [31] developed gold conjugated MNPs, as shown in Figure 3b. The results exhibited that the aptamers bound with specific receptors present on the surface of prostate cancer cells or in the neovasculature of tumors. Meanwhile, Brero et al. [111] reported a combination of carbon ion and hyperthermia therapy applied to pancreatic adenocarcinoma cells, for assessing the antitumor efficiency. They also proposed protocols for future clinical applications, which were compared to an investigation using a 6 MV photon beam irradiation jointly to hyperthermia. On the other hand, Umut et al. [112] investigated ^1^H nuclear magnetic resonance (NMR) relaxometry to indicate the suspensions of NiFe_2_O_4_ required for superparamagnetic MRI contrast agents. They reported the presence of agglomerates, and particularly interactions within the agglomerated MNPs, resulting in a significant increase in the herthermia and MRI efficiencies.

A prime example of peptides is the amino acid HIV-1 Tat protein, which has been conjugated to gold nanoparticles, resulting in rapid intracellular uptake and localization to the nucleus [33]. Taratula et al. [34] reported the enhanced reduction in cell viability by using hormone-release hormone (LHRH) peptide coated MNPs, as shown in Figure 3c. Niemirowicz et al. [113] reported an increase in the anticancer activity of MNPs by the surface attachment of CLL-37. They treated colon cancer cells (i.e., DLD-1 cells and HT-29 cells) with MNPs. CLL-37 showed a decrease in cell viability compared with CLL-37 free MNPs. Cathelicidin (CLL-37) and chlorotoxin are two peptides known to bind with, and inhibit the proliferation of, cancer cells [35]. 

Although targeting of cancer cells is a popular aspect, limited MNP dosage delivery to tumor cells through intravenous administration indicates a need for intratumoral delivery of MNPs [18]. Tumor cells are highly heterogeneous, as the composition of stroma and parenchyma cells and the type/ratio of secreted proteins vary greatly with the type of tumor. Generally, it is suggested that smaller MNPs go deeper into the tumor extracellular matrix, while larger sized MNPs are restricted to the immediate vicinity of the vascular extravasation [18].

## 5. Clearance of MNPs

After intravenous injection, MNPs must avoid rapid clearance by four organs: liver, kidney, bone marrow, and spleen. The mode of clearance is characteristically dependent on the properties of MNPs, such as size, shape, and charge chemistry. The size and surface properties of nanoparticles also affect their overall distribution in the tumor area, retention time, and excretion rate [114]. MNPs larger than 100 nm in size are discharged through the body very quickly. Hence, smaller particles are more favored. However, smaller particles possess a higher surface area to volume ratio, which favors protein adhesion. MNPs adhered to protein molecules can be recognized easily by macrophages and cleared through the spleen and liver. Hence, surface modifications are needed to get the optimum size of MNPs, and surface coatings are required to get stability and a balance between the size of MNPs and their retention time within the body [8]. The size of MNPs also affects their ability to extravagate from the bloodstream to tumor tissues through the transcytosis process [115]. 

Surface charge is another parameter that influences the circulation rate, retention time, and ultimately heating performance. The positive charge allows the MNPs to adhere nonspecifically to the cell membrane, while the negative charge causes adsorption of plasma proteins, and thus enhances their uptake by macrophages, reducing their circulation time in blood [116]. Similarly, hydrophilic and neutral MNPs show little interaction with plasma protein, and thus show an enhanced heating efficiency due to longer circulation time [117]. 

Shape is another factor determining the biodistribution of MNPs. Differently shaped nanoparticles interact differently with protein corona, which affects the elimination rate. Muro et al. [118] developed elliptical discs and their counter spherical particles. Elliptical discs showed a longer circulation time. Decuzzi et al. [119] reported that oblate spheroids showed a longer circulation time than spherical nanoparticles. Devarajan et al.’s [120] results demonstrated a superior accumulation of work and rod-like nanoparticles in tumor tissues compared with counter spherical nanoparticles. Eliezar et al. [121] developed folate decorated worm-like and spherical nanoparticles for drug delivery. Worm-like particles showed a higher accumulation rate in major organs, i.e., liver, kidney, and spleen. However, several other factors affect the biodistribution of nanoparticles and their accumulation, such as high intra-tumor pressure, presence of dense matrices within the tumor cells, and heterogeneous tumor vascular cells. 

## 6. Typical Examples of Magnetic Hyperthermia with MNPs

**Figure 4:**
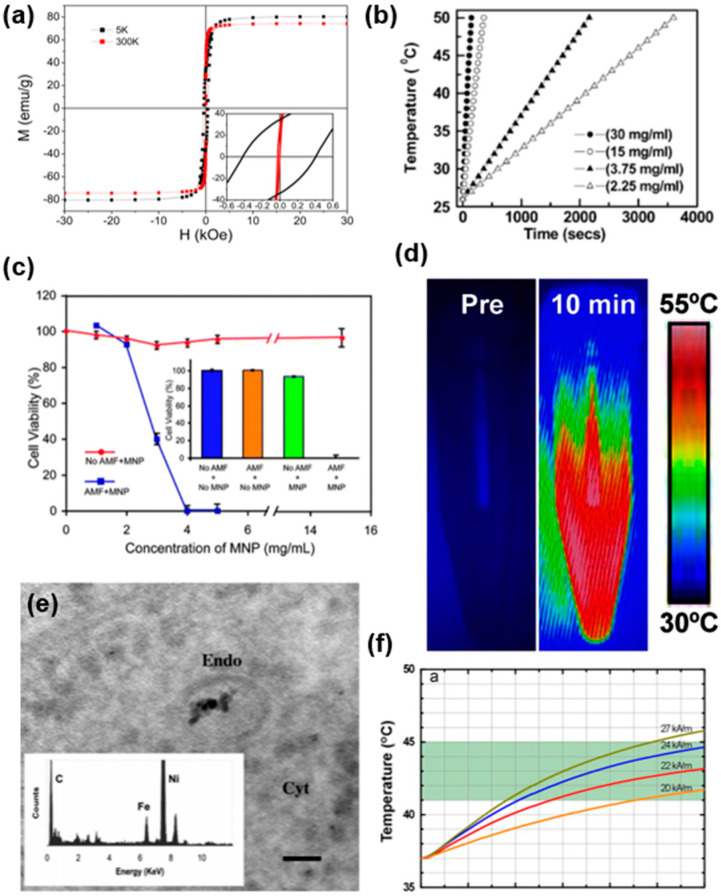
(**a**) Magnetization values of iron oxide MNPs at different field strengths, 5 K (black) and 310 K (red). Inset shows the low field region to estimate the coercive field. Reprinted with permission from [83]. Copyright 2012 American Chemical Society. (**b**) Heat generation of iron oxide base MNPs at 6.5 kA/m magnetic field and 60 kHz. Reprinted with permission from [122]. Copyright 2007 The Royal Society of Chemistry. (**c**) Cell viability of Hela cells using protein-coated iron oxide MNPs. Reprinted with permission from [124]. Copyright 2008 The Royal Society of Chemistry. (**d**) Thermal heating of FA-PEG-SPION MNPs. Reprinted with permission from [126]. Copyright 2013 Ivyspring International. (**e**) TEM image, showing agglomeration of MNPs in an endosome in KB cells and inset showing the EDX analysis. Reprinted with permission from [127]. Copyright 2005 American Chemical Society. (**f**) Heating profile of Fe/MgO under different alternating magnetic field strengths. Reprinted with permission from [131]. Copyright 2011 Elsevier.

Iron oxide MNPs have attracted great attention in magnetic hyperthermia due to their strong magnetization properties, as shown in Figure 4a [83]. Table 3 shows an overview of a typical example of MNPs in magnetic hyperthermia. Prasad et al. [122] investigated the heat generation of iron-based MNPs in deionized water when exposing the material to an external magnetic field of 6.5 kA/m strength, at a frequency of 60 kHz. A profound increase in temperature of the water suggested significant magnetic heating properties, as shown in Figure 4b. Furthermore, they also investigated the increment in heat generation with the increase in the concentration of MNPs. Interestingly, the MNPs showed a saturation point of heat generation, named Curie temperature. A further increase above Curie temperature was not achieved, even with a further enhancement in MNP concentration. 

Jerry et al. [123] developed protein passivated iron oxide nanoparticles with low inherent cytotoxicity. The developed particles displayed a significant killing of cancerous cells, and the cell viability tremendously decreased with the increase in the concentration of MNPs from 1–4 mg/L, as shown in Figure 4c. In another study, Hayashi et al. [124] prepared cysteine-modified iron oxide nanoparticles by the hydrolysis-condensation method. The material showed excellent magnetic hyperthermia, with a SAR of 156 W g^−1^. Recently, Le Renard et al. [125] investigated the magnetic heating properties of iron oxide MNPs embedded in a silica shell by recording the specific power loss values (20 W g^−1^) under an alternating magnetic field of 141 kHz, 12 mT. 

Hayashi et al. [126] investigated the magnetic heating properties of folic acid (FA) and polyethylene glycol (PEG) modified clusters of superparamagnetic iron oxide nanoparticles (SPIONs), as shown in Figure 4d. The magnetic heating was investigated by preparing the suspension of material into water and then exposing it to an AC magnetic field of 8 kA/m strength at 230 kHz. The increase in the temperature of the solution was ≈20 °C for 10 min. Furthermore, the developed MNPs were injected into mice bearing subcutaneous xenograft tumors. After twenty-four hours, decreases in magnetic resonance imaging (MRI) signal intensity and enhanced image contrast were recorded in regions enclosed in the red dotted line. During MRI, the T_2_ value of cancer cells was found to decrease with time, but that of muscle was unchanged. Furthermore, a histological analysis proved that the synthesized particles resided in cancer cells, not necrotic tissues. The biodistribution of synthesized particles in major organs, including the tumor, was assessed by the nitroso-PSAP method at 24 h post-injection. The uptake of nanoparticles was reported in the following order: cancer cells > spleen > liver > kidney. In another study, Sonvico et al. [127] showed the uptake of MNPs by TEM images, as shown in Figure 4e. EDX spectroscopy also confirmed the presence of MNPs within the tumor.

Maier-Hauff et al. [128] successfully treated fourteen patients with brain tumors using amino-silane-coated SPIONs. These patients received multiple treatments. The maximum temperature attained by cancer cells during each treatment was 42.4–49.5 °C. No significant toxicity was observed. Furthermore, significant advances in magnetic hyperthermia occurred when the antibody-attached SPIONs were developed. DeNardo et al. [129] studied the effects of antibody-tagged, dextran- and PEG-coated SPIONs in hyperthermia treatment of nude mice. It is worth noting that the applied fields used were quite large, at 700, 1000, or 1300 Oe (56, 80, or 104 kA/m). Nanoparticles had no observable toxicity. Jimbow et al. [130] proposed another SPIONs system, and the melanogenesis substrate, N-propionylcysteaminylphenol, was selectively incorporated into melanoma cells and inhibited their growth by the production of cytotoxic free radicals.

Although iron oxide nanoparticles are of high magnetization values they suffer from chemical stability and biocompatibility issues [132]. Recently, Chalkidou et al. [131] used Fe particles coated with biocompatible MgO, providing satisfactory chemical stability and biocompatibility. The results showed a significant decrease in cancerous cell viability, while normal cells remained same compared with control samples, along with profound heat generation under variable alternating magnetic field strengths (Figure 4f). In addition, drug release has been recognized as another promising application of MNPs which exhibit temperature-dependent slow drug release behavior. As a typical example, a cell apoptosis assay presented that doxorubicin-ferumoxytol-medical chitosan nanoparticles could provide synergistic efficacy in colon carcinoma cell treatment in vitro compared to other therapeutic groups. Heat induction in mice subcutaneous xenografted tumors demonstrated the better heating performance of such MNPs [133,134].

**Table 3:** Overview of typical examples of magnetic hyperthermia.

No.	Compound	Preparation Method	Alternating Magnetic Field Strength	Magnetic Properties		Frequency	Cell Line	Ref.
				Ms	Hc			
			Am^−1^	emu/g	Oe	kHz		
1	Fe_3_O_4_	Coprecipitation	6.3	N/A	N/A	400	Hela cells	[123]
2	Fe_3_O_4_	Spray-coprecipitation	150	25.6	58	64.6	KB and L929 cancer cells	[135]
3	Fe_3_O_4_	One pot hydrolysis condensation reaction	N/A	24	0	230	Glioma 261	[124]
4	Fe_3_O_4_	Coprecipitation	26.6 *	59.7	100	265	MCF7 human breast cancer cells	[136]
5	Fe_3_O_4_	Thermal decomposition	29	80	0	520	KB cancer cells	[83]
6	Fe_3_O_4_	Coprecipitation		16	284	~750–1150	HFL1 cells	[137]
7	Fe_3_O_4_	N/A	12.7 *	68	N/A	250	Dendritic cells	[138]
8	*γ* − Fe_2_O_3_	Coprecipitation	88 **	N/A	N/A	108	MCF7, KB 3-1, HeLa cell line	[127]
9	*γ* − Fe_2_O_3_	Massart method	N/A	200	N/A	100	A549 cells, Saos-2 cells HeLa cells, and HepG2 cells	[139]
10	*γ* − Fe_2_O_3_	Sol–gel	12 **	2.5	3.44	141	N/A	[125]
11	Fe/MgO	Vapor condensation	8–29 *	210	N/A	765	MCF7 and MDA-MB231 breast cancer cell	[131]
12	*γ* − Mn_0.2_Fe_1.8_ O_3_	Thermal decomposition	100 ***	78	<10	425	HeLa cell	[122]
13	*γ* − Mn_x_Fe_2- x_O_3_	Thermal decomposition	150 ***	25–78	N/A	425	HeLa cell	[140]
14	ZnGd_0.02_Fe_1 0.98_O_4_	Coprecipitation	6.5	N/A	N/A	60	VE Cells	[141]

Note: * values are in kAm^−1^; ** values are in mT; *** values are in Oe; N/A: Not Available.

## 7. Summaries of Requirements in Hyperthermia with MNPs

Thermal ablation based on MNPs has a remarkable potential for application in cancer treatment. Recent developments in controlling the size, shape, composition, and coating material of MNPs have led to the establishment of a large tool box to engineer the physio-chemical properties of MNPs, which will enable efficient heat performance and high SAR values. This review presented an overview of magnetic hyperthermia using MNPs with different heating capabilities and thermal doses. It also covered a brief introduction of the MNP synthesis techniques and coating materials that have been developed to help accomplish magnetic hyperthermia therapy. Extremely versatile and multi-functional MNPs can be synthesized by combining the favorable properties of MNPs with polymer functionality. Despite tremendous improvements in the synthesis and characterization of MNPs, the field of magnetic hyperthermia still faces several challenges. For instance, the heating performance of MNPs must initially be evaluated to establish their performance in cell ablation. In several examples in Section 6, it has been shown that, due to protein corona formation, the heating performance is diminished and evaluation by using immobilized MNPs is required. Another major challenge regards the heating performance of MNPs, which is related with the parameters of the applied magnetic field during the experiments, such as frequency and strength of magnetic field. Targeting cancerous cells is one more major obstacle. Functional moieties-conjugated MNPs are beneficial in this aspect. The complexity of the MNPs involved with cancer cells is also very important in comparing the performances of different MNPs. Finally, the MNPs are delivered in lower dosages compared with the expected values in intravenous administration.

It is important to use superparamagnetic MNPs to reduce the aggregation in the body after magnetic heating treatment, while the surface coating of MNPs can also reduce aggregation. MNP concentration can be intensified by intratumoral administration, instead of intravenous shots. In principle, magnetic hyperthermia by MNPs is capable of efficient cancer treatment. However, more in-depth work is needed to better explain the biological mechanisms and nanoscale heating, from a single-cell level to the whole body.

## Figures and Tables

**Table 2:** Commonly used polymers for magnetic nanoparticle coating.

No.	Material	Advantage	Ref.
1	Polyethylene glycol	Improve the biocompatibility of the NPs by resisting protein adsorption and increasing their intracellular uptake	[96]
2	Polyvinyl alcohol	Monodisperse particles are formed with reduced coagulation	[97]
3	Dextran	The stable colloidal suspension is formed along with enhanced blood circulation time	[98]
4	Chitosan	Produce biocompatible and hydrophilic particles	[99]
5	Polyacrylic acid	Produce biocompatible and stable particles	[100]
6	Polyvinylpyrrolidone	The stable colloidal suspension is formed along with enhanced blood circulation time	[101]
7	Poly(D, L-lactide)	Biocompatible and low cytotoxicity	[102]
8	Gelatin	Biocompatible and hydrophilic particles are formed	[103]
